# Focusing on Social Behaviors: Improving the Perceived Warmth of Sharks in an Aquarium Setting

**DOI:** 10.3390/ani13152455

**Published:** 2023-07-29

**Authors:** Joao Neves, Jean-Christophe Giger, Vasco Alves, Nuno Soares

**Affiliations:** 1 Department of Science and Education, Zoomarine Algarve, 8201-864 Albufeira, Portugal; 2 Psychology Research Centre (CIP), University of Algarve, 8005-139 Faro, Portugal; 3 Faculty of Human and Social Sciences, University of Algarve, Campus de Gambelas, 8005-139 Faro, Portugal

**Keywords:** sharks, warmth, conservation, stereotype content model, aquarium

## Abstract

**Simple Summary:**

Today, around one-quarter of all shark species are threatened with extinction, and action is needed to reverse this trend. Several factors are known to influence conservation efforts, among which is their negative perception, which influences the way people perceive and emotionally engage with these top predators. Aquariums worldwide play a strong role in this strategy as they host millions of visitors every year and, for many of these, are one of the only chances to see sharks up close and aim to create an everlasting positive perception of these fish. This study tested two different communication strategies to best engage visitors in shark awareness and conservation when visiting an aquarium with sharks. Our results show that, by exposing visitors to the unknown social behaviors of sharks with the help of an interpreter (educator), their perception of these animals slightly improved. Other findings on the potential use of videos as attention grabbers are considered.

**Abstract:**

Sharks are commonly depicted as intentionally dangerous predators and are considered a threat by the general public, limiting support for and success of global shark conservation. Following the SCM framework, this study aimed at testing the effect of information on the social lives of sharks alone or paired with circumstantial humor on the participants’ perceived warmth of sharks before visiting an aquarium. The present study took place in a naturalistic setting, allowing testing of the variables in a pseudo-real-world environment where results can objectively help in the implementation of strategies on the ground. A total sample of 303 visitors participated in this study, where three conditions (control: 100; social information: 102; social information with humor: 101) were tested. Results showed that, although mild, it was possible to affect the warmth dimension of the shark’s stereotype, most likely due to the presence of information about the social lives of sharks. This information slightly leveraged the perceived warmth dimension, although still far from the less threatening stereotype as aimed. Results also highlight the possible importance of using videos within the strategic communication and education approaches in aquariums in order to be most effective in challenging the shark stereotype. Limitations and future research ideas are explored.

## 1. Introduction

A growing number of shark species are cataloged as endangered, according to the International Union for Conservation of Nature (IUCN). By 2009, Dulvy et al. reported that around 24% of shark species were considered to be endangered by the IUCN Red List [1]. More recently, Pacoureau and colleagues (2021) showed a dramatic 71% decrease in the world population of sharks and ocean rays since 1970 [2]. Due to an 18-fold increase in fishing pressure throughout the last decades, 75% of all sharks and rays are at risk of extinction [2]. Threats such as by-catch fishing or habitat destruction are playing a role in the future of shark populations [2], but public perception of sharks is also one of the main concerns regarding their conservation. In fact, within the marine environment, sharks are one of the species most vilified by the general public [3]. Indeed, when asking randomly selected adults about their least liked animals, Stephen Kellert (1985) found that sharks ranked in the top 10 [4]. Popular entertainment has also greatly reinforced avoidance emotions, which has been particularly detrimental to sharks. For example, Australian political actors used movie narratives to influence risk management measures after shark accidents [5]. By analyzing political decisions for risk management after shark accidents, the author found that many political speeches were aligned with cinematic narratives instead of scientific evidence. A recent study by Le Busque and Litchfield (2022) analyzed 109 shark movies and concluded that the sharks are portrayed in a similar way to how these animals are portrayed in the news media as a potential threat to humans [6]. Indeed, sharks are commonly depicted as intentionally dangerous predators [7,8] and are considered a threat by the general public [9]. This negative public image of sharks has been linked with overestimated shark-attack risks [10] and public fear [11], thus limiting support for and success of global shark conservation efforts (e.g., [6,12,13,14,15]).

### 1.1. Stereotyping Sharks

**Figure 1:**
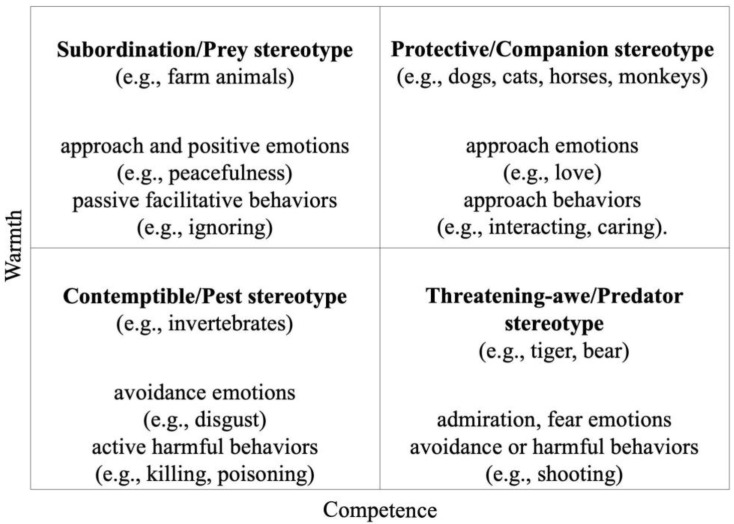
The Stereotype Content Model (SCM) applied to animals. Table adapted from [24]. The stereotypes and affective emotions and behaviors result from the combinations of perceived warmth and competence.

According to Cardwell, a stereotype is “... a fixed, often simplistic generalization about a particular group or class of people” [16] (p. 227). Through stereotyping, we infer that an individual has a set of characteristics and skills that other members of his group also have. Stereotypes lead to social categorization, which is one of the reasons for the creation of prejudices, leading to processes of aggregation (associated with the ingroup) or segregation (associated with the outgroup). Fiske and colleagues (2002) first proposed the Stereotype Content Model (SCM), postulating that group stereotypes arise from two major dimensions: warmth and competence [17]. These have precedents in other theoretical frameworks, perhaps the most relevant being Agency and Communality, in the context of psychology and religion [18], as well as in impression formation [19]. The general principle of the SCM is based on the characteristics that we believe a social object has and will perhaps be the most important factor in establishing our main perception of that object. Of special relevance, warmth, and competence are two separate dimensions, conceptually orthogonal. As such, a low or high result in one of these dimensions can be combined with a high or low result in the other dimension. Warmth encompasses “traits that are related to perceived intent, including friendliness, helpfulness, sincerity, trustworthiness and morality” [20] (p. 77). Competence encompasses “traits that are related to perceived ability, including intelligence, skill, creativity and efficacy” [20] (p. 77). Extending the SCM, Cuddy et al. (2007) then proposed the Behaviors from Intergroup Affect and Stereotypes (BIAS) map [21]. With this approach, combinations of perceived warmth (low vs. high) and competence (low vs. high) were associated with different types of stereotypes, influencing emotions (approach vs. avoidance emotional states) and intergroup behaviors (active vs. passive, facilitative vs. harmful). As people routinely attribute human characteristics (e.g., traits, intentions, emotions) and stereotypes to animals using anthropomorphism, human–animal relationships are influenced. Recently, Sevillano and Fiske (2016, 2019) have suggested that the SCM/BIAS map framework could also be applied to animals to describe and explain these relationships [22,23]. These authors found that, of 25 animals studied, each one could be fitted into one of four stereotypes, mimicking those of human intergroups [24] (see Figure 1).

In a recent study, Neves et al. (2021) found that sharks are stereotyped, following the same social cognition traits as with stereotyping humans [8]. In fact, sharks were depicted as stereotypically more masculine (i.e., more agentic/independent than communal). This gendered perception is also consistent with sharks being associated with avoidance emotions. These authors also found sharks, within the Agency vs. Communality dimensions, as less communal (warm) and highly agentic (competent) animals [8]. Following this stereotypical perception of sharks, the relationship between the perception of sharks’ communality, conservation attitudes, and behavior was then studied by Neves et al. (2021) [25]. Results showed a clear dominance of competence over the warmth dimension, clearly fitting within the ambivalent threatening-awe stereotype. This stereotype involves some prejudice toward subjects, as they are feared due to some traits of high competence (e.g., aggressiveness, dominance) but also hold our attention and admiration due to other traits such as beauty, intelligence, or determination. Neves and colleagues (2021) also found that both perceived warmth and competence were associated with specific emotions and attitudinal and behavioral tendencies, which, in turn, could predict positive attitudes and behaviors toward conservation [25]. Approach emotions like dazzlement and excitement predicted positive attitudinal and behavioral tendencies toward sharks. The perception of warmth was also shown to have an indirect positive effect on attitudes toward conservation using the associated approach emotions [25]. The perception of sharks as competent animals also seemed to relate to positive attitudes towards shark conservation [25].

### 1.2. Increasing the Warmth Dimension

Following these thoughts, the authors point to the idea that increasing the perception of warmth toward sharks may also increase public support for shark conservation. Specifically, drawing attention to some traits commonly dissociated from sharks, such as sociability, collaboration, or protection, could enhance their perceived warmth. In fact, evidence shows that the perception and manipulation of warmth traits affect society’s active involvement in other social issues. Lee et al. (2018) examined the effects of warmth and competence in sports teams when partnering with other organizations (e.g., government and nonprofit organizations) and how these combined perceptions affected individuals’ willingness to donate to corporate social responsibility initiatives [26]. While they discovered that emphasizing partnerships high on warmth would benefit the overall perception of the sports organization, the authors highlighted the importance of communication in cases of low-warmth partners (such as sharks, for the purpose of this study). They highlighted the significance of using specific words, phrases, and images that underline positive warm traits. In fact, Li et al. (2020) studied the neural representation of warmth and competence and found that, after reading two successive trait-implying behavioral descriptions, participants did not completely interpret and represent warmth and competence traits as independent dimensions [27]. Warmth was also found to be more influential than competence traits. A possible explanation is that warmth is a more important, broad, and dominant dimension in social interaction and cooperation.

All in all, our view of sharks may be difficult to change due to the perception of sharks as highly competent animals, but also due to cultural and popular beliefs. The continuous portrayal of sharks as blood-driven killing machines creates a conflicting impression that sharks are a threat to humans when they should be seen otherwise. Adding to this, the sharks’ physical appearance and predator-like traits provide them with a high status and level of competitiveness. These two traits together actively influence the perceived low warmth and high competence of this animal, challenging any strategy to enhance their ‘good side’.

### 1.3. Humor as a Persuasion Tool

Humor has been used as emotional leverage in advertising, being used as a tool for consumer persuasion [28]. It is also suggested that humor has the unique quality to overcome resistance to persuasive messaging, raise awareness, and encourage positive attitudes and behaviors while simultaneously minimizing conflict, anger, and resistance [29]. In a study using humor in advertising, Strick and colleagues (2012) found humor to be both a distractor and a motivator of liking and approach behavior, thus breaking the resistance to persuasion [28]. Since more arousing stimuli are remembered better than less arousing ones [30], humor works like other arousing emotional experiences (e.g., fear), directing cognitive attention toward those stimuli (e.g., predators) at the expense of less arousing stimuli.

Currently, scholars agree on three prevailing theories related to humor appreciation [31]. The Incongruity Theory proposes that humor arises from the perception of incongruity or a violation of expectations, leading to surprise and cognitive dissonance [32]. The Superiority Theory proposes that humor stems from a sense of superiority over others, involving mockery or derision towards individuals or situations that are seen as inferior, which elicits amusement and laughter [33]. Lastly, the Relief Theory proposes that humor provides a release of tension or anxiety, allowing individuals to discharge nervous energy and reduce stress [33]. It is this theory that guides the present research, aiming to use humor solely as a distractor to promote pleasant situations [34], relieve anxiety, increase positive affection [33], and increase the communicator’s credibility and acceptance [35,36]. In short, humor can facilitate empathy and reinforce social bonds, features deeply related to the warmth dimension.

### 1.4. Objectives

Following the SCM, the current study aimed at testing whether exposing novel social information about sharks alone or paired with circumstantial humor would positively influence the perceived shark’s warmth in a natural setting experiment.

We sought to test the following research hypotheses:

**H1.**  *By drawing attention to some traits commonly dissociated from sharks, such as sociability, collaboration, or protection, information about the social lives of sharks increases the perception of the warmth of these animals.*

**H2.**  *By employing circumstantial humor as a distractive technique to enhance positive emotions, along with providing details about the social lives of sharks to promote the credibility and acceptance of the message, the perception of sharks’ warmth can be improved.*

## 2. Materials and Methods

### 2.1. Participants and Design

The methodology took place in a natural setting (aquarium of Zoomarine Algarve, Portugal) and involved a sample of adult visitors. This naturalistic approach allows testing the variables in a pseudo-real-world environment where the results can objectively help in the implementation of strategies on the ground.

303 adult randomly assigned zoo visitors voluntarily participated in this study. We manipulated participants’ exposure to content about sharks and compared responses across three independent conditions (control: 100 participants; social information: 102 participants; social information with humor: 101 participants). One condition was tested per day. Days per condition were randomly assigned. The experiment ran for a total of 48 days. Visitors, on average, spent 11 min from the entrance hall to the survey point area, close to the exit.

Participation followed the ethical standards for research on humans as required by the host institution. Subjects were informed about their rights to participate and the possibility to stop at any moment with no repercussions. An informed consent form was signed by all participants, stating that all data would be treated holistically and that the ethical principles of confidentiality and anonymity would be respected. All procedures in this study followed the APA ethical principles and Portuguese regulations about data protection.

### 2.2. Procedure and Instruments

Control condition: Groups of fifty zoo visitors were welcomed to the aquarium’s entrance hall, where they waited 5 min to enter the circuit. During this period, visitors were told that the wait time was just so the aquarium would not become too crowded. No additional information was provided about the visiting experience.

Social Information Condition (henceforth named Informational): As with the control condition, visitors were welcomed to the aquarium’s entrance hall. With the support of a TV, sound system, and PowerPoint presentation, an educator explained some of the species and habitats available when visiting the aquarium. The narrative was focused on the biological and ecological characteristics of the aquarium species with no reference to sharks. At the end of the interpretation, a video (2:25 min) was played whose content addressed the social characteristics of sharks. No humor was used with the information provided.

Social Information with Humor Condition (henceforth named Informational with Humor): The sole difference from the Social Information Condition was the active use of circumstantial and non-shark-related humor and interaction with the waiting visitors. The narrative had a mix of fun facts and biological traits of the aquarium species. At the end of the interpretation, the same video about the social life of sharks was screened. No mention of sharks was ever made by the educator.

The video used images showing some of the social characteristics of sharks. The narrative (Portuguese voiceover and English subtitles) was created to reinforce the shark’s social characteristics, as well as use human behavior analogies to increase empathy. The video was created in a newsflash format to promote credibility (see Video Script S1 in Supplementary Material).

Close to the exit of the aquarium, a random sample of adult visitors were asked to answer a questionnaire based on Neves et al. (2021), assessing the participants’ perception of sharks, perceived femininity, attitudes toward conservation, approach–avoidance emotions, and donation intentions [25] (see Supplementary Material). To ensure random allocation, every third adult crossing a predetermined invisible line was asked to answer the survey. No information about the study was given to the visitors before answering the survey. To ensure that the two conditions could not overlap, each was delivered across the entire day. All scales displayed good internal reliability (Table 1). Participants were asked about their gender, age, animal interest, and occupation.

### 2.3. Preliminary Analysis and Control Checking

Skewness and kurtosis values were analyzed, and all values were below the threshold recommended by Curran et al. (1996) [37] (i.e., 2 and 7, respectively), and thus, normality assumptions were met.

### 2.4. Data Analysis

A total of eight outliers were identified and removed from the final data (Control: 6; Informational: 1; Informational with Humor: 1). A one-sample t-test between the middle point of the scale (3.5) for warmth and competence to test if average scores were significantly different from neutrality. One-way ANOVA with Tukey HSD post hoc tests were used for comparing multiple samples. A GLM analysis was performed for the covariates of gender, age, and animal interest. Pearson correlations were also performed to test for the linear relationship between variables. Analysis was performed using IBM SPSS Statistics, version 23 (IBM Corp., Armonk, NY, USA).

## 3. Results

### 3.1. Overall Descriptive Analysis

Out of the total participants, 54% (163) identified as female, while 46% (140) identified as male. The majority, 69.5%, reported being Portuguese, while the remaining 30.5% identified as foreigners. On average, participants rated their animal interest as generally high (*M* = 6.28; *SD* = 1.06), with no differences found between conditions, *F* (2,284) = 2.83, *p* = 0.60, $\eta_{p}^{2}$ = 0.02. The participants, on a scale of 0 to 100 for the perception of masculinity, rated the shark with an average score of 57.5 (*SD* = 15.94), differing significantly from the middle point of the scale (*t* (278) = 7.86, *p* < 0.001). No significant differences between conditions were found, F (2,276) = 1.60, *p* = 0.20, $\eta_{p}^{2}$ = 0.011.

### 3.2. Stereotypes of Sharks

Both warmth and competence for the control condition showed significant differences (*p =* 0.015; *p* < 0.001, respectively), confirming the threatening-awe stereotype (low warmth and high competence) previously found. Participants also found sharks markedly masculine, reaffirming the gendered stereotype (Table 1).

### 3.3. Effect on Shark’s Stereotype

**Figure 2:**
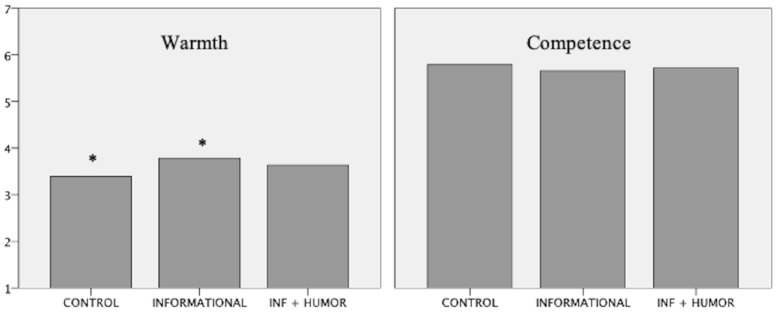
Warmth and Competence scores between conditions (Warmth—* with significant differences (Tukey HSD *p* < 0.05)).

We found significant differences in warmth between Control and Informational conditions (*p* < 0.05) and no differences for Informational with Humor condition, *F* (2,292) = 3.149, *p =* 0.044, $\eta_{p}^{2}$ = 0.021 (Figure 2). No differences across conditions were found for the competence dimension, *F* (2,292) = 1.158, *p =* 0.286, $\eta_{p}^{2}$ = 0.009 (Figure 2). No significant differences were found in any of the covariates. No differences were found between conditions for any of the other dimensions measured (intention to donate, attitudes towards conservation, approach–avoidance emotions; all *p* > 0.05).

### 3.4. Correlation Analysis

Although we found correlations across almost all dimensions measured within the Control condition, with mostly weak effects, only two slightly moderate correlations were found between approach emotions and competence/attitudes towards conservation (*r* = 0.399, *p* < 0.01/*r* = 0.428, *p* < 0.01) (Table 2).

Similarly, the Informational condition showed a similar weak to the moderate associative pattern between variables (Table 3).

Even though no differences were found with any other condition, the use of circumstantial humor and directing attention to the social behavior of sharks showed correlations with all dimensions measured, although mostly weak. No association effect was found between the two stereotypical dimensions (warmth and competence) (Table 4).

Between conditions, competence/avoidance emotions correlation showed a significant change with the inclusion of circumstantial humor, shifting from neutral to a weak negative effect. Correlation between competence/intention to donate also increased with both Informational and Informational with Humor conditions. Warmth was also more strongly correlated with the intent to donate in both conditions when compared to the Control condition. The correlation between avoidance emotions and intention to donate showed a negative effect with the inclusion of circumstantial humor.

## 4. Discussion

This study tested whether exposing social information about sharks alone or paired with circumstantial humor would positively influence the perceived shark’s warmth. With regard to our first research hypothesis, i.e., exposing information about the social lives of sharks increases the warmth dimension of these animals, our results showed a slight increase in warmth between the Control and Informational conditions (Figure 2). This positive effect responds positively to our initial objectives, but we have to consider two factors at play: the physical presence of an educator and the video on the social behavior of sharks. From the methodological standpoint, there is no way of directly selecting the influence of one over the other, but considering the growing amount of evidence on the comfort of video screening and retaining attention [38,39], we suspect that the presence of the video played a key role in the present study’s findings. Looking at the participants’ ages and understanding what draws millennials’ attention [38,39], the appeal of the video fits quite well. If this is indeed the case, the absence of influence in the Informational with Humor condition can be associated with a distractor effect. We argue that, since humor preceded the video screening, it may have worked as a distractor to the video message. The video may not be engaging enough to catch visitors’ full attention, and therefore, it is not guaranteed that the information is retained, as it has been previously shown in past research (for a review, see [40]).

Our results, however, did not confirm our second research hypothesis, i.e., pairing circumstantial humor with information about the sharks’ unknown social lives improves their perceived warmth. Warren et al. (2019) have found similar negative results with brand attitudes and humor [41]. By analyzing the use of humor in advertisements, these authors found that humor may, in fact, increase negative emotional reactions toward brands.

Generally speaking, no structural change was observed in any of the dimensions under study. Nevertheless, circumstantial humor seemed to have a mildly negative influence on the correlation between avoidance emotions and the intention to donate.

To our best knowledge, no other studies have yet tried to improve the perceived warmth dimension of sharks using either social information or humor. Our findings, although not conclusive, shed some light on some interesting facts that may help guide future communication strategies for shark conservation, with specific applications in real-world settings such as zoos and aquariums.

### 4.1. The Social Lives of Sharks

Our results show that, although mild, it was possible to affect the warmth dimension of the shark’s stereotype, most likely due to the presence of information on the unknown social lives of sharks. This information slightly leveraged the perceived warmth dimension about sharks, although not enough to the less threatening stereotype as aimed. Although our approach cannot be considered training, as the exposure to the information of the unknown social lives of sharks was very brief, the use of this information may have worked as a sort of counter-stereotype, as it does not negate the stereotype per se but rather activates positive and unrelated associations from memory [30]. Since sharks are not usually perceived as social creatures or as having parental care (a set of features strongly associated with warmth), by conveying this information, participants were not exposed to contradictory stereotypical information but rather to a different set of characteristics usually unrelated to its stereotype. This exposure worked to activate mostly positive information, as it relates to our own perception of warmth, and never by negating the common perception of competent animals. In fact, increased knowledge has been shown to positively influence conservation support [42]. This approach has been shown to be effective in reducing automatic stereotype activation and, thus, possible negative evaluations [43].

### 4.2. The Naturalistic Setting

We set out to try to increase the warmth perception of sharks in a naturalistic setting, such as an aquarium because it fits particularly well with the premise for this research: aquariums are, par excellence, education places where people go of their own volition, often on a family trip, and are open to getting information and learning about animals (cognition) [44]. It’s also possible to see living sharks first-hand, giving visitors the chance to emotionally experience their presence (emotion). Without any information given beforehand, some aquariums may be stereotype maintainers, as our results show with the control condition. With the presence of an educator and the screening of a video on the social lives of sharks, we could identify a slight improvement in the warmth dimension. These results give us useful information for future research and a good ground to rethink the communication paradigm for these animals, tackling their solid and stable threatening-awe stereotype.

### 4.3. The Video Effect

In a final remark, our results also shed some light on the importance of integrating short videos within the strategic communication and education approaches at aquariums. Zoos and aquariums today are mostly visited by two distinct cohorts (GenX and Millennials) with the ability to politically and socially influence the immediate conservation of sharks [45]. With a growing number of Millennials choosing to visit these places as they reach parenthood, the need arises to adapt to their communication strategy as well as their education approach in order to be most effective in challenging the shark stereotype.

### 4.4. Limitations and Future Research

While these studies provide some interesting insights into a possible strategy to improve the shark stereotype, some limitations have been identified throughout the research. We found that many external factors came into play and affected our results. The absence of sound changes between conditions, i.e., a visible increase in the warmth dimension, can be associated with the experimental design, where participants spent an average of 11 min between exposure to the condition and answering the survey. This prolonged time, added to the presence of many distracting elements (different aquariums and interaction with family members and other visitors), may have also conditioned results. The experiment also did not study the individual effect of the educator’s presentation vs. the screening of the shark social information video. This would more clearly determine the real influence of exposure to social information about sharks vs. the presence of an educator on the shark stereotype. One last limitation relates to the fact that there was no induction check regarding whether each condition had a different emotional influence on participants.

## 5. Conclusions

We sought to explore whether exposing aquarium goers to social information alone or with circumstantial humor could be used to leverage the warmth perception of sharks. Although the present study did not find a sound positive influence on the shark stereotype, it nevertheless showed a mildly positive influence by adding information about the unknown social behaviors of sharks. The distracting effect of using circumstantial humor paired with the social information did not report any significant change in the shark stereotype. This study also points to some possible paths to explore in conservation education programs in zoos and aquariums.

## Figures and Tables

**Table 1:** Descriptive Statistics.

	Control			Informational			Inform. with Humour			
	M	SD	N	M	SD	N	M	SD	N	α
Competence	5.99 *	0.59	94	5.84	0.68	101	5.91	0.67	100	0.71
Warmth	3.40 *	0.97	94	3.79	1.17	101	3.64	1.13	100	0.88
Avoidance	3.25	1.38	94	3.34	1.32	99	3.38	1.29	95	0.87
Approach	4.51	1.30	94	4.70	1.11	99	4.53	1.08	96	0.88
Conservation	6.23	0.95	94	6.25	0.99	99	6.22	0.99	95	0.78
Donation	2.68	0.86	85	2.54	0.81	89	2.68	0.79	84	
Masculinity	56.1 *	13.9	92	56.2 *	15.6	93	59.6 *	17.7	88	

Note. * = Mean scores differ significantly at *p* < 0.05 from the middle point of the scale (e.g., 3.5 for all scales except masculinity’s middle point = 50).

**Table 2:** Correlations found with the Control Condition.

	1	2	3	4	5	6
1. Competence	1	0.260 *	0.052	0.399 **	0.241 *	0.058
2. Warmth		1	−0.146	0.273 **	0.091	−0.045
3. Avoidance			1	−0.213 *	−0.274 **	0.153
4. Approach				1	0.428 **	−0.241 *
5. Conservation					1	−0.299 **
6. Donation						1

Notes: * *p* < 0.05; ** *p* < 0.01.

**Table 3:** Correlations found with the Informational Condition.

	1	2	3	4	5	6
1. Competence	1	0.372 **	0.051	0.358 **	0.125	−0.229 *
2. Warmth		1	0.039	0.359 **	0.133	−0.327 **
3. Avoidance			1	−0.224 *	0.002	0.158
4. Approach				1	0.288 **	−0.347 **
5. Conservation					1	−0.238 *
6. Donation						1

Notes: * *p* < 0.05; ** *p* < 0.01.

**Table 4:** Correlations found with the Informational with Humor Condition.

	1	2	3	4	5	6
1. Competence	1	0.182	−0.284 **	0.386 **	0.247 *	−0.216 *
2. Warmth		1	−0.276 **	0.393 **	0.298 **	−0.361 **
3. Avoidance			1	−0.323 **	−0.309 **	0.413 **
4. Approach				1	0.377 **	−0.425 **
5. Conservation					1	−0.265 *
6. Donation						1

Notes: * *p* < 0.05; ** *p* < 0.01.

## Data Availability

Data sharing is not applicable to this article due to privacy and ethical restrictions.

## Supplementary Materials

The following supporting information can be downloaded at: https://www.mdpi.com/article/10.3390/ani13152455/s1, S1: Video Script—Social Lives of Sharks; S2: Questionnaire—Shark stereotype.
