# Supplementary material for: Focusing on Social Behaviors: Improving the Perceived Warmth of Sharks in an Aquarium Setting
Source: Animals (Basel). 2023 Jul 29;13(15):2455. doi: 10.3390/ani13152455 (PMC10416988; doi:10.3390/ani13152455)
Supplement: Supplementary file 1 [file animals-13-02455-s001.zip › animals-2490955-supplementary.pdf]

S1. Script used for the newsflash video on the social life of sharks

Sharks are much more social than we think and therefore very different from their reputation as solitary animals.

Recent studies suggest that, in addition to society's image of these fish, many sharks enjoy the company of family and friends.

Researchers are just beginning to understand that these animals, after all, have complex social lives, worthy of a prime-time soap opera.

By aggregating into schools, establishing alliances and other links with each other, they ensure joint benefits in hunting or protection, helping to explain why many of these fish not only tolerate each other, but also often seem to enjoy doing so.

Among the more than 400 species of sharks, there are some that go out to lunch together in certain estuaries and it is even suspected that the location of preferred feeding areas is socially transmitted between individuals and generations.

It's so interesting that sharks seem to share information about the best feeding places, perhaps in a marine and less technological version of TripAdvisor.

Many sharks are also seen swimming together, often with a preference for a particular companion.

Even though our perception tells us that sharks are solitary predators, we now know that many species gather in large numbers in well-known areas of the ocean, to hunt or simply socialize.

Some schools of sharks can reach tens of individuals. There are others, such as hammerhead sharks, which can gather in groups that easily number in the hundreds.

Lemon sharks, which live in mangroves, estuaries and reefs, never seem to be alone. They form bonds with other lemon sharks, in addition to being frequently accompanied by remoras, in a close relationship almost as intense as that of a marriage.

There are also records of some species in which groups of pregnant females gather in bays and other areas of shallow waters, demonstrating protective behaviors often observed in other species usually more empathetic to us.

S2. Questionnaire

Please classify the SHARK according to the following criteria / For me the SHARK is:

	**Nothing**						**A lot**
Friendly	1	2	3	4	5	6	7
Competent	1	2	3	4	5	6	7
Well-intentioned	1	2	3	4	5	6	7
Trustworthy	1	2	3	4	5	6	7
Warm	1	2	3	4	5	6	7
Confident	1	2	3	4	5	6	7
Independent	1	2	3	4	5	6	7
Competitive	1	2	3	4	5	6	7
Capable	1	2	3	4	5	6	7
Kind	1	2	3	4	5	6	7
Efficient	1	2	3	4	5	6	7
Sincere	1	2	3	4	5	6	7
Tolerant	1	2	3	4	5	6	7
Attentive	1	2	3	4	5	6	7
Skillful	1	2	3	4	5	6	7
Intelligent	1	2	3	4	5	6	7
Amicable	1	2	3	4	5	6	7
Empathetic	1	2	3	4	5	6	7
Creative	1	2	3	4	5	6	7
Smart	1	2	3	4	5	6	7

When I see / think of a SHARK I feel…

	**Nothing**						**A lot**
Uncomfortable	1	2	3	4	5	6	7
Cheerful	1	2	3	4	5	6	7
Dazzled	1	2	3	4	5	6	7
Happy	1	2	3	4	5	6	7
Accomplished	1	2	3	4	5	6	7

Horrified	1	2	3	4	5	6	7
Disgusted	1	2	3	4	5	6	7
Confident	1	2	3	4	5	6	7
Angry	1	2	3	4	5	6	7
Curious	1	2	3	4	5	6	7
Afraid	1	2	3	4	5	6	7
Worried	1	2	3	4	5	6	7
Excited	1	2	3	4	5	6	7
Threatened	1	2	3	4	5	6	7
Enthusiastic	1	2	3	4	5	6	7

In your opinion:

**More efforts should be made to protect sharks**								
Strongly disagree	1	2	3	4	5	6	7	Strongly agree
**Conservation of sharks is a matter for humans**								
Strongly disagree	1	2	3	4	5	6	7	Strongly agree
**Shark conservation is the responsibility of human**								
Strongly disagree	1	2	3	4	5	6	7	Strongly agree

Quantify, in percentage, how masculine and feminine the shark is by dividing 100%:

The shark is _______% feminine and %________ masculine
(write directly the % in the open spaces - the total has to make 100%)

If you had the opportunity to donate to various conservation organizations, indicate the order from the most important (1) to the least important (4): conservation trust for a) crocodiles; b) turtles; c) sharks; d) dolphins.

- Crocodiles ( )
- Turtles ( )
- Sharks ( )
- Dolphins ( )

Tell us a bit about you:

Age: ________ Gender: _________ Job: ______________________

Nationality: ( ) Portuguese ( ) Other

Mother tongue: ( ) Portuguese ( ) Other

Place of origin (where you live for most part of your life): __________________________

Are you fond of animals?

Not at all	1	2	3	4	5	6	7	A lot
